# Crohn's Colitis Care, a Disease‐Specific Electronic Medical Record, Enhances Data Capture in Pediatric Inflammatory Bowel Disease Care

**DOI:** 10.1002/jgh3.70153

**Published:** 2025-04-30

**Authors:** Joseph Louis Pipicella, Shoma Dutt, Kunal Thacker, Susan Jane Connor, Jane Mary Andrews, Angharad Vernon‐Roberts

**Affiliations:** ^1^ University of New South Wales, Medicine & Health (South Western Sydney Clinical School) Sydney New South Wales Australia; ^2^ Ingham Institute for Applied Medical Research, Gastroenterology, Hepatology and Inflammatory Bowel Disease Research Group Liverpool New South Wales Australia; ^3^ Crohn's Colitis Cure Sydney New South Wales Australia; ^4^ Department of Gastroenterology The Children's Hospital at Westmead, Sydney Children's Hospitals Network Westmead New South Wales Australia; ^5^ The University of Sydney Children's Hospital Westmead Clinical School Sydney New South Wales Australia; ^6^ Liverpool Hospital Department of Gastroenterology and Hepatology Liverpool New South Wales Australia; ^7^ Central Adelaide Local Health Network Adelaide South Australia Australia; ^8^ University of Adelaide, Faculty of Health Sciences Adelaide South Australia Australia; ^9^ Department of Paediatrics University of Otago Christchurch Christchurch New Zealand

**Keywords:** colitis, Crohn disease, electronic health records, inflammatory bowel diseases, outpatients, pediatrics

## Abstract

**Background and Aim:**

Crohn's Colitis Care, a structured, disease‐specific electronic medical record, is proven to promote more complete data capture in adult Inflammatory Bowel Disease care. This study aimed to determine whether similar effectiveness was seen in pediatrics.

**Methods:**

Matched patient records from a hospital's standard electronic medical record (pre‐Crohn's Colitis Care) and those in Crohn's Colitis Care were retrospectively reviewed (12 months each). The presence of disease‐specific data items per platform were compared (21 core, 5 age‐specific). Data are presented as percentage recorded (recorded items/total eligible for age). Descriptive and statistical analytics were used.

**Results:**

Paired records were reviewed for 114 children, of whom 78 (68%) had Crohn's disease and 69 (61%) were male. Median age at diagnosis was 13.5 years (IQR12.0–15.5), with mean disease duration 3.6 years (±2.4). Crohn's Colitis Care was more likely to capture 9 items: general wellbeing, stool urgency and frequency, disease duration, comorbidities, pubertal stage, sexual activity, alcohol and drug usage (each *p* < 0.05). The standard platform was more likely to capture 4 items: liquid stools, phenotype, disease indices, and vaccinations (each *p* < 0.05). Crohn's Colitis Care achieved more eligible data items recorded per patient (75.3% ±11.5 vs. 67.7% ± 8.9; *p* < 0.001). Item completion rate in both platforms inversely correlated with patient age (*p* < 0.05).

**Conclusions:**

Consistent with findings in adult care, Crohn's Colitis Care achieved more complete disease‐data capture in pediatrics compared to a standard platform. Given that not all items were better recorded in the structured platform, work to understand and address barriers is needed to optimize complete data capture for care and research.

## 1 Introduction

Around 1 in 290 Australians live with an inflammatory bowel disease (IBD), namely Crohn's disease (CD) or ulcerative colitis (UC) [1, 2], with these numbers rapidly increasing [3, 4]. People with active disease may experience diarrhea, abdominal pain, stool urgency, and rectal bleeding, as well as other functional, nutritional, and psychological comorbidities [5]. People with IBD exhibit a reduced propensity to engage in regular physical activity [6] and are also known to experience anxiety‐depressive disorders and sleep quality issues, even during periods of disease remission [7]. Notably, around 10% of people are diagnosed prior to 18 years of age, necessitating lifelong care [8]. The management of IBD is complex and generally requires a multidisciplinary approach [9]. However, research suggests current IBD care models are inadequate, inconsistent, and inequitable [10, 11, 12].

Research also shows documentation of clinical information and quality measures to be poor across multiple IBD clinics [13, 14]. Electronic medical records (EMRs) should improve workflow efficiencies and access to comprehensive patient data to enable complex, multidisciplinary care [15, 16]. However, 41% of clinicians are unconvinced they have timely access to reliable electronic patient records, and 35% of patients are concerned that their treating team does not have access to their relevant health data [17]. EMRs have also been associated with increased documentation time and physician burnout [18, 19], with some suggesting the inclusion of patient‐entered information as a potential solution [20].

While EMRs have digitized data recording, there is an absence of automation and standardization [16], and most healthcare data is captured in a free‐text format. Calls have been made for more structured and standardized documentation to increase EMR value and enable easy and accurate ‘data reuse’ for quality measurement purposes, scientific research, and decision support without the need for extensive downstream re‐processing, which can lead to errors, diminished data granularity [21, 22, 23] and add significant wasteful emissions [24]. In 2015, the co‐design of an IBD‐specific, cloud‐based, outpatient EMR called Crohn's Colitis Care (CCCare) commenced [25, 26]. Today, CCCare is used for routine care documentation by over 300 clinicians across 22 IBD care sites in Australia and New Zealand to document routine care including clinical assessments and management plans. It is also used by people with IBD to complete pre‐clinic questionnaires based on general well‐being and symptom burden via a linked consumer portal. Structured data entered into CCCare subsequently flow into a secure, de‐identified clinical quality registry for audit and research. The structured nature of these data enable reuse with little downstream processing and no loss of granularity [27].

CCCare is proven to be effective in promoting more complete IBD‐specific data capture in adult care [28], but whether the same holds true in pediatric care remains unknown. The aim of this study was to determine the effectiveness of CCCare at improving clinically relevant data capture for pediatric people with IBD compared to a standard, free‐text EMR.

## 2 Methods

### 2.1 Study Design

This was a single center, observational, and retrospective comparative study performed at The Children's Hospital at Westmead, New South Wales, Australia.

### 2.2 Ethics Statement

This study was ethically approved by the South Western Sydney Local Health District Human Research and Ethics Committee (HREC) (2021/ETH11378).

### 2.3 Participants

All children with IBD aged 0–18 years with a documented outpatient clinical encounter in both the standard, free‐text EMR and CCCare were included.

### 2.4 Data Retrieval

This study did not collect any specific data from children or clinicians. For all children, demographic data derived from either EMR were recorded, including sex, age, body mass index, residential location, whether English was their second language, and/or whether they required an interpreter. Disease‐related variables recorded in either EMR were also recorded, including disease diagnosis and duration, and disease activity status. A patient was defined as in remission if they had a fecal calprotectin of < 250 μg/g [29] or a disease activity index score (Pediatric Crohn's Disease Activity Index [PCDAI] [30] or Pediatric Ulcerative Colitis Activity Index [PUCAI] [31]) of ≤ 10 during the 12 months of data collection per EMR.

### 2.5 EMR Platforms

The standard EMR referred to in this study was PowerChart version 2018.01 (2018. Kansas City, MO, US: Cerna Corp), which is a predominately free‐text, hospital‐wide platform used for viewing patient results and documenting clinical encounters. CCCare is an IBD‐specific EMR platform containing predominately structured data collection fields with some free text fields available. Structured data collection in CCCare is formatted as drop‐down lists, checkboxes, and radio buttons.

### 2.6 Data Dictionary

A data dictionary containing IBD‐specific items considered to be essential for holistic pediatric care (derived from the CCCare‐pediatric co‐design consultation process) [23] was created (Table 1). The presence of each item within each EMR was scored and compared.

**TABLE 1:** Data dictionary containing IBD‐specific items.

Topic area	IBD‐specific items
Well‐being	General Well‐Being
	Days Out of Role (E.g. School, Work or Sport)
	Limitations To Activity
	Abdominal Pain
Stool metrics	Stool Urgency
	Rectal Bleeding
	Stool Consistency
	Liquid Stool
	Daily Bowel Frequency
	Bowel Frequency Compared To ‘Normal’ or Baseline
	Nocturnal Bowel Frequency
Recreational activities	Smoking Status
	Alcohol Consumption
	Sexual Activity
	Recreational Drug Use
Disease‐specific variables	Disease Phenotype (Paris Classification)
	IBD‐Related Surgeries
	Disease Duration or Date of Diagnosis
	Disease Activity Index (PCDAI or PUCAI)
	Extra Intestinal Manifestations
	Comorbidities
	Adherence To IBD Therapy
Growth	Height
	Weight
	Tanner Stage of Puberty
Vaccination	Vaccinations

Some items were age dependent. The Tanner Stage of Puberty [32] was sought during data review if the participant was ≥ 12 years [33], whereas recreational activity variables (smoking status, alcohol use, sexual activity and recreational drugs) were only sought during data review in those aged ≥ 14 years [31, 34, 35].

### 2.7 Data Retrieval Process

For each participant, all ambulatory data entries into the standard EMR (including scanned images of hand‐written notes) recorded within the 12 months prior to CCCare implementation were reviewed. In CCCare, all entries made up to 12 months post‐implementation were reviewed. Medical record reviews were conducted by a single researcher (JLP) for consistency, and the presence or absence of each item within the data dictionary was documented per patient for each EMR.

### 2.8 Outcomes

- Primary outcome:
  ○ the overall completeness of IBD data per patient in each EMR.
- Secondary outcomes:
  ○ the completion of specific, pre‐defined, IBD data items per patient in each EMR
  ○ the factors associated with the overall completeness of IBD‐specific data documentation in both EMRs.

### 2.9 Analysis

Descriptive statistics were used for demographic variables, presented as number (%) and median (interquartile range [IQR]). The overall total percentage of variables completed was derived according to the age of each participant, then compared between the two EMRs using a paired sample t‐test (after confirming data were normally distributed). For age‐dependent variables, percentages were derived according to the relevant denominator determined by participant age.

For each individual variable, the percentage completed in the standard EMR and CCCare were compared using McNemar's two‐proportion z test. Analysis of factors associated with total percentage was compared using analysis of variance for categorical data, and linear regression for continuous variables. The significance level was set at *p* < 0.05 for all analyses. Data were analyzed using SPSS version 29.0 (2022. Armonk, NY, US: IBM Corp).

## 3 Results

### 3.1 Demographics

A total of 114 children with IBD had paired standard EMR and CCCare medical records reviewed (Table 2).

**TABLE 2:** Cohort demographics of children with IBD.

Variable	*N* (%)
Total number of patients reviewed	114 (100)
Male Sex	69 (61)
Median Age, years (IQR)	
At Diagnosis	11.0 (9.0–13.0)
At Start of Standard EMR Period	13.5 (12.0–15.5)
Diagnosis	
CD	78 (68)
UC	34 (30)
IBDU	2 (2)
Median Disease Duration, years (IQR)	3.1 (1.9–4.8)
Median BMI, kg/m^2^ (IQR)	20.4 (17.4–23.2)
English as a Second Language	26 (23)
Interpreter Required	8 (7)
Residential Region	
Metropolitan area	106 (93)
Small regional center	7 (6)
Large regional center	1 (1)

Abbreviations: BMI, Body Mass Index; CD, Crohn's Disease; EMR, Electronic Medical Record; IBDU, Inflammatory Bowel Disease Unclassified; IQR, Interquartile Range; UC, Ulcerative Colitis.

### 3.2 Overall Data Completion

CCCare recorded more IBD‐specific data items per patient (75.3% ±11.5, range 13.6–92.3) compared to the standard EMR (67.7% ±8.9, range 34.6–90.9) (*p* < 0.001, CI −9.8 to −5.4).

### 3.3 IBD‐Specific Item Completion in CCCare

CCCare was significantly more likely to capture data on general well‐being, stool urgency, bowel frequency compared to normal, nocturnal bowel frequency, disease duration, comorbidities, Tanner Stage of Puberty, alcohol, recreational drug use, and sexual activity (Table 3). Vaccinations were recorded for just 9% of children in CCCare (Table 3).

**TABLE 3:** Comparison of proportions completed for each variable per patient in each EMR.

Topic	Variable	Denominator (N)	Standard EMR *N* (%)	CCCare *N* (%)	*p* value	95% CI
Well‐being	General Well‐Being	114	87 (76)	112 (98)	< 0.001	−0.30 to −0.14
	Days Out of Role	114	106 (93)	111 (97)	0.13	−0.10 to 0.01
	Activity Level	114	106 (93)	110 (97)	0.21	−0.09 to 0.02
Stool metrics	Abdominal Pain	114	114 (100)	110 (97)	0.05	0.00 to 0.07
	Urgency	114	11 (10)	113 (99)	< 0.001	−0.95 to −0.84
	Rectal Bleeding	114	113 (99)	111 (97)	0.32	−0.02 to 0.05
	Stool Consistency	114	113 (99)	112 (98)	0.56	0.02 to 0.04
	Liquid Stool	114	110 (97)	98 (86)	0.005	0.04 to 0.18
	Bowel Frequency Compared to Normal	114	10 (9)	113 (99)	< 0.001	−0.96 to −0.85
	Daily Bowel Frequency	114	112 (98)	111 (97)	0.66	−0.03 to 0.05
	Nocturnal Bowel Frequency	114	61 (54)	109 (96)	< 0.001	−0.52 to −0.32
Recreational^a^	Smoking Status	47	0 (0)	43 (92)	0.05	0.01 to 0.17
	Alcohol Consumption	47	0 (0)	6 (13)	< 0.001	0.78 to 0.97
	Sexual Activity	47	0 (0)	5 (11)	< 0.001	0.81 to 0.98
	Recreational Drug Use	47	0 (0)	6 (13)	< 0.001	0.78 to 0.97
Disease – Specific Variables	Phenotype	114	90 (79)	41 (36)	< 0.001	0.32 to 0.54
	Disease Activity Index (PCDAI/PUCAI)	114	109 (96)	58 (51)	< 0.001	0.36 to 0.54
	Disease Duration	114	106 (93)	112 (98)	0.03	−0.10 to 0.01
	EIMs	114	97 (85)	104 (91)	0.14	−0.14 to 0.02
	Comorbidities	114	62 (54)	103 (90)	< 0.001	−0.46 to −0.26
	IBD‐Related Surgeries	114	21 (18)	21 (18)	1.0	−0.10 to −0.10
	Adherence to IBD Therapy	114	30 (26)	21 (18)	0.16	−0.03 to 0.19
Growth	Height	114	114 (100)	111 (97)	0.08	0.00 to 0.06
	Weight	114	114 (100)	111 (97)	0.08	0.00 to 0.06
	Tanner Stage of Puberty^b^	96	27 (28)	55 (57)	< 0.001	−0.42 to −0.17
Other	Vaccination	114	88 (77)	10 (9)	< 0.001	0.59 to 0.78

Abbreviations: CI, Confidence Interval; CCCare, Crohn's Colitis Care; EIM, Extra Intestinal Manifestation; IBD, Inflammatory Bowel Disease; PCDAI, Pediatric Crohn's Disease Activity Index; PUCAI, Pediatric Ulcerative Colitis Activity Index.

^a^ Reviewed only in those aged ≥ 14.

^b^ Reviewed only in those aged ≥ 12.

### 3.4 IBD‐Specific Item Completion in Standard EMR

The standard EMR was significantly more likely to capture data on liquid stools, disease phenotype (Paris Classification [36]), a disease activity index, and vaccinations (Table 3). Recreational items (smoking, alcohol consumption, sexual activity and recreational drug use) were never captured in the standard EMR, and bowel frequency compared to normal was captured in only 9% (Table 3).

### 3.5 Factors Associated With Overall IBD Data Documentation

**FIGURE 1:**
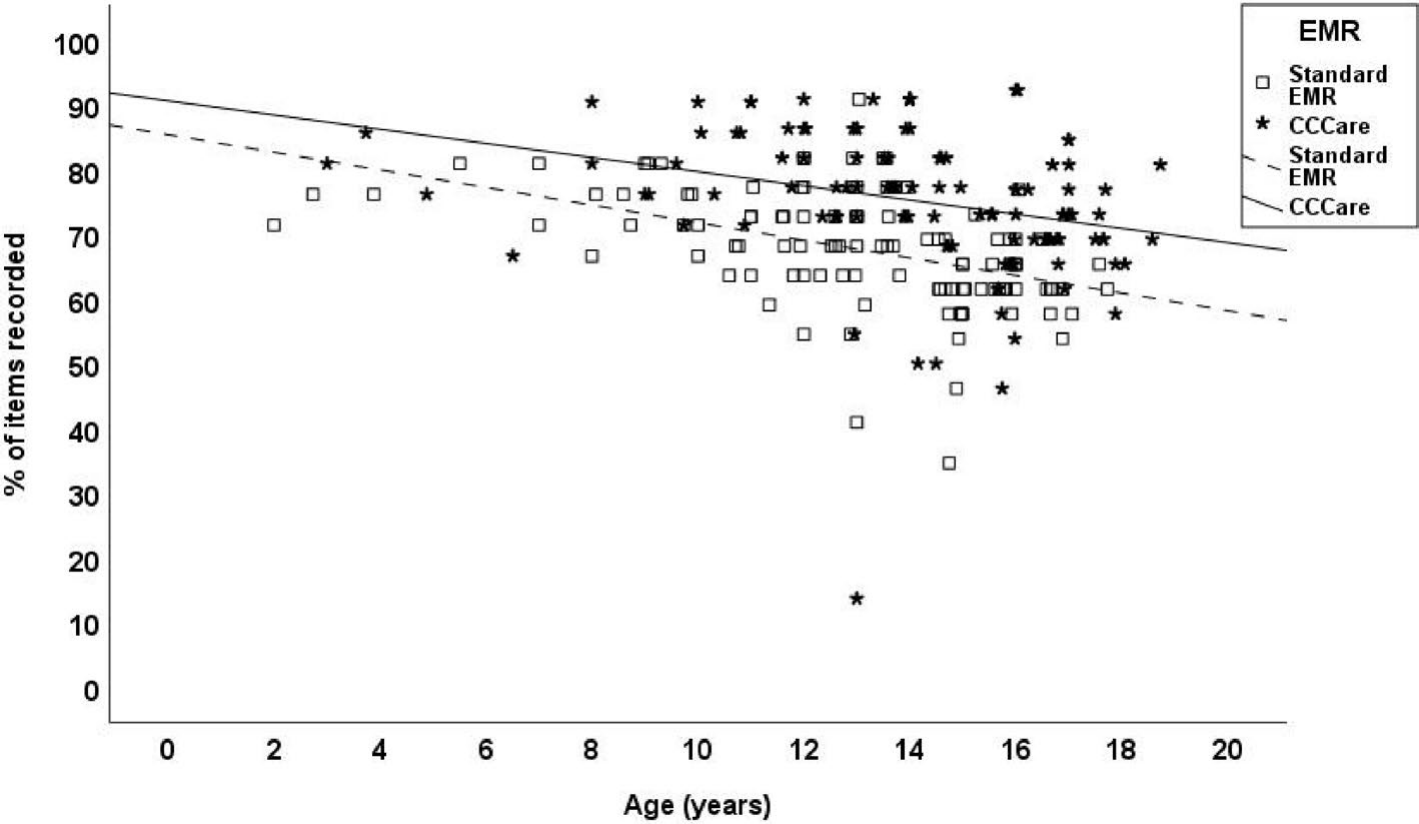
Percentage of Disease‐Specific Items Recorded by Age. CCCare, Crohn's Colitis Care; EMR, Electronic Medical Record.

Few variables were associated with overall data completeness in either EMR; however, item recording in both was inversely associated with patient age, with the number of items recorded per patient decreasing as children grew older (*p* ≤ 0.002) (Table 4, Figure 1). The completing clinician did not influence the degree of data recorded within the standard EMR nor CCCare (Table 4).

**TABLE 4:** Factors explored for association with item completion rate per EMR platform.

Variable	Standard electronic medical record			Crohn's Colitis Care		
	Mean diff	*p* value	95% CI	Mean diff	*p* value	95% CI
Biological Sex	−0.03	0.99	−3.4 to 3.4	0.10	0.96	−4.3 to 4.5
Disease Diagnosis^a^	1.4	0.44	−2.2 to 4.9	2.3	0.32	−6.9 to 2.3
Residential Area^b^	2.2	0.49	−4.2 to 8.7	2.1	0.62	−6.2 to 10.5
English as Second Language	−0.08	0.69	−4.7 to 3.2	−1.1	0.67	−6.2 to 4.0
Disease Remission	2.6	0.14	−0.9 to 6.1	−0.34	0.89	−5.0 to 4.3
Completing Clinician	N/A^c^	0.15	−0.2 to 0.1	N/A^c^	0.05	−0.02 to 0.1
	**R**	***p* value**	**95% CI**	**R**	***p* value**	**95% CI**
Age	0.46	0.001	−1.8 to −0.9	0.29	0.002	−1.8 to −0.4
Duration Disease	0.02	0.84	−0.8 to 0.6	0.028	0.77	−1.0 to 0.8

Abbreviations: CI, Confidence Interval; Diff, Difference; *p* value, Probability value; R, Correlation Value.

^a^ Crohn's Disease versus Ulcerative Colitis/Inflammatory Bowel Disease Unclassified.

^b^ Metropolitan vs. Regional (Small/Large).

^c^ Mean difference marked as not appropriate (N/A) as the analysis of variance included more than two groups.

## 4 Discussion

This study compared the degree of IBD‐specific data capture between a standard EMR and CCCare at a tertiary, Australian pediatric IBD clinic. Overall, CCCare recorded more IBD‐specific data items per patient compared to the standard EMR, suggesting that time‐poor clinicians may document a greater level of clinically relevant data when using a structured EMR with built‐in prompts and reminders. This may ultimately lead to better‐informed clinicians.

Contrary to the findings of Weiskopf et al. [34] who found ‘sicker’ patients (not specifically with IBD) receiving anesthesia were more likely to have greater data recorded in their EMR compared to ‘healthier’ patients, our study found no relationship between the number of items collected and remission status. The current study also found that the number of items collected per patient decreased with age. This differs from a study that looked at data completion in children with human immunodeficiency virus, where those of a younger age (0–14 years) were found to have a larger proportion of missing data items compared to older patients [35]. This could be attributed to the fact that these studies were conducted in different countries on different diseases. Notably, older age and greater time since diagnosis is associated with lower parental involvement in IBD care [37], and symptom reporting is known to differ between children with IBD and their parents (with parents more likely to over‐estimate their child's symptoms) [38].

In the current study, CCCare was significantly more likely to capture recreational data around alcohol consumption, drug use, and sexual activity (in those aged ≥ 14 years). Similar to one study that found sex and other adult issues were not discussed with patients' pre‐transition to adult care settings [39], these items were never recorded in the standard EMR. This is concerning, given the mean age of first alcohol consumption and illicit drug use in Australia is 16.2 and 17.3 years respectively [33], and both alcohol consumption and illicit drug use have the potential to worsen disease activity and outcomes [40, 41]. Similarly, the median age at first sexual intercourse in Australia is 16 years, with some children having intercourse as early as 12 years of age [42, 43]. Contrarily, CCCare was more likely to document sexual activity compared to the standard EMR in children aged ≥ 14 years, although overall numbers were still low (11% in CCCare vs. 0% in standard EMR). Guidelines suggest that doctors discuss sexual activity with all patients capable of reproduction [44, 45], particularly in those receiving potentially teratogenic therapies such as methotrexate [46]. The standard EMR similarly failed to capture any data on smoking in children aged ≥ 14 years, despite data showing the mean age Australian's first smoke a full cigarette is 16 years [31]. This is important, given smoking has been shown to worsen CD activity [47].

It has been known for quite some time that recreational topics are rarely discussed with pediatric patients [35], and this current study found that in the standard EMR, this was still not documented by clinicians. Therefore, a system to drive consistent collection of such data is needed. CCCare's ability to better capture recreational data could be explained by its structured fields, which may prompt clinicians to enquire during consultations, or by its consumer portal, which allows children to self‐report, from their own devices, on information that they may otherwise be uncomfortable discussing face‐to‐face with their doctors, particularly with their parent/s in the room.

Active disease and mucosal inflammation can impact growth and puberty onset. Studies show around 10% of children with IBD experience delays in puberty onset, with up to 40% experiencing growth retardation [48, 49]. In Australia, approximately 80% of all 12–13‐year‐olds have commenced puberty [50], yet only 28% of children aged ≥ 12 years in the current study had a Tanner Stage of Puberty recorded in their standard EMR, while 57% had their Tanner Stage recorded in CCCare, a significantly greater number. This may be explained by CCCare's inclusion of an embedded staging chart for reference during consultations.

The standard EMR was significantly more likely to capture data on disease activity indices, phenotype, and vaccinations. This could be explained by CCCare's reliance on clinicians to manually enter data, some of which would generally be automatically recorded in other standard EMR platforms. For example, the standard EMR platform in this study had results from pathology tests performed within the hospital, such as hematocrit, erythrocyte sedimentation rate, and albumin (required for PCDAI calculation) automatically recorded for clinicians. There are plans for this functionality to be built into CCCare as part of the iterative development process. Similarly, given the standard EMR was not disease specific and was used hospital‐wide, general health items like vaccinations or height and weight could be recorded by clinicians from varying disciplines, increasing documentation likelihood. Further training for clinicians using CCCare to improve familiarity with data fields within the platform, as well as integration of CCCare with pathology providers and/or nation‐wide medical records and immunization registries, would improve the amount of data recorded in CCCare, thus addressing this deficit. In both platforms, adherence to IBD therapies was infrequently recorded. This is of particular importance, given medication non‐adherence has been shown to be closely correlated with higher disease activity [51].

### 4.1 Strengths

This study explored a broad number of IBD‐specific data items and topics across a relatively broad age range in matched patient records. It is the only study that compares real‐world data capture between CCCare and a standard EMR platform in a pediatric cohort.

### 4.2 Limitations

Some assumptions have been made regarding age‐related items, with some children below the threshold potentially being missed in these analyses. Different time periods for data collection per EMR may have also impacted the types or degree of data collection. As an example, endoscopic fields could not be accurately compared due to the small number of children that had an endoscopy performed within both the standard EMR and CCCare data review periods. Furthermore, psychosocial assessments could not be compared given the standard EMR did not have any functionality to distribute these to patients as CCCare did. This study was retrospective and limited to one center, with only a select number of clinicians entering data, which may have introduced bias. An increased sample size would have made results more generalizable.

## 5 Conclusions

Consistent with findings in adult care, CCCare facilitates greater disease‐data capture compared to a standard platform in pediatrics. Clinicians will therefore have a greater awareness of patients' disease and wellbeing, improving outcomes and expediting comprehensive communication within the multidisciplinary team. Given that not all items were better recorded in the structured platform, work to understand and address barriers is needed to optimize complete data capture for care and research.

## Ethics Statement

This study was ethically approved by the South Western Sydney Local Health District Human Research and Ethics Committee (HREC) (2021/ETH11378).

## Consent

The study site was responsible for obtaining consent from patients or their parents prior to their data being stored in the Crohn's Colitis Care (CCCare) platform. For more information, see https://c‐c‐cure.org/privacy‐policy/.

## Conflicts of Interest

Joseph Louis Pipicella is Crohn's Colitis Cure (CCCure) Employee & Company Secretary within study duration. Conference Funding: Dr. Falk Pharma, Ferring Pharmaceuticals. Shoma Dutt Funds for Advisory Boards Received from: Dr. Falk and AbbVie. Clinical Advice given to the Australian Pharmaceutical Benefits Advisory Committee (PBAC) (no funds received). Kunal Thacker – Nil. Susan Jane Connor is Honoraria for Advisory Board participation, speaker fees, educational support and/or research support from: Abbvie, Amgen, BMS, Celltrion, Chiesi, DrFalk, Eli‐Lilly, Ferring, Fresenius Kabi, Gilead, GSK, Janssen, MSD, Organon, Novartis, Pfizer, Sandoz, Takeda, Vifor, Agency for Clinical Innovation, Gastroenterological Society of Australia, Medical Research Future Fund (2021–25), South Western Sydney Local Health District (2018–2023), Sydney Partnership for Health, Research and Enterprise (SPHERE) and The Leona M and Harry B Helmsley Charitable Trust (2020–2026). Other executive memberships: Member of ANZIBD consortium (current); Executive committee member of ANZIBDC/CCA Research Priorities Committee (in the last 3 years). SONIC Health shareholder. SWSLHD employee. Jane Mary Andrews, Speaker's fees, research support, Ad Boards, industry consultation: AbbVie, Allergan, Anatara, Atmo Capsule, Bayer, BMS, Celgene, Celltrion, Falk, Ferring, Fresenius Kabi, Gilead, Hospira, Immuninc, ImmunsanT, Janssen, MSD, Nestle, Novartis, Pfizer, Sandoz, Shire, Takeda, Vifor, RAH research Fund, The Hospital Research Fund 2020–2022, The Helmsley Trust 2020–2026. Digital health demonstration project with NOVARI via CALHN. CALHN employee. Board Chair CCCure. Director of GESA until Sept 2023. Vernon‐Roberts, Angharad: Nil.

## Data Availability

Data, analytic methods, and study materials will not be made available to other researchers except on specific request.
